# Supplementary material for: Stigma and Emotion Regulation in Intimate Partner Violence: A Pilot Exploratory Study with Victims, Offenders and Experts
Source: Behav Sci (Basel). 2025 Sep 10;15(9):1229. doi: 10.3390/bs15091229 (PMC12466727; doi:10.3390/bs15091229)
Supplement: Supplementary file 1 [file behavsci-15-01229-s001.zip › behavsci-3821373-supplementary.pdf]

Questionnaires

Victims' group

n° - role investigated	Italian	English (translation)
1 - victim	*Si trova coinvolto/a in una situazione di violenza di genere nel ruolo della vittima: come si descrive?*	*You find yourself involved in an episode of gender-based violence in the role of victim: how do you describe yourself?*
2 - victim	*Si trova coinvolto/a in una situazione di violenza di genere nel ruolo della vittima: l'aggressore come la descrive?*	*You find yourself involved in an episode of gender-based violence in the role of victim: how does the offender describe you?*
3 - offender	*Si trova coinvolto/a in una situazione di violenza di genere nel ruolo della vittima: come descrive l'aggressore di tale episodio?*	*You find yourself involved in an episode of gender-based violence in the role of victim: how do you describe the offender in such episode?*

Offenders' group

n° - role investigated	Italian	English (translation)
1 - offender	*Si trova coinvolto/a in una situazione di violenza di genere nel ruolo dell'aggressore: come si descrive?*	*You find yourself involved in an episode of gender-based violence in the role of offender: how do you describe yourself?*
2 - offender	*Si trova coinvolto/a in una situazione di violenza di genere nel ruolo dell'aggressore: la vittima come La descrive?*	*You find yourself involved in an episode of gender-based violence in the role of offender: how does the victim describe you?*
3 - victim	*Si trova coinvolto/a in una situazione di violenza di genere nel ruolo dell'aggressore: come descrive la vittima di tale episodio?*	*You find yourself involved in an episode of gender-based violence in the role of offender: how do you describe the victim of such episode?*

Experts' group

n° - role investigated	Italian	English (translation)
1 - victim	*Si trova coinvolto/a in una situazione di violenza di genere nel ruolo della vittima: come si descrive?*	*You find yourself involved in an episode of gender-based violence in the role of the victim: how do you describe yourself?*
2 - victim	*Si trova coinvolto/a in una situazione di violenza di genere nel ruolo dell'aggressore: come si descrive?*	*You find yourself involved in an episode of gender-based violence in the role of offender: how do you describe yourself?*
